# Attitudes towards Social Networking and Sharing Behaviors among Consumers of Direct-to-Consumer Personal Genomics

**DOI:** 10.3390/jpm3040275

**Published:** 2013-10-14

**Authors:** Sandra Soo-Jin Lee, Simone L. Vernez, K.E. Ormond, Mark Granovetter

**Affiliations:** 1 Stanford Center for Biomedical Ethics, 1215 Welch Road, Mod A, Stanford, CA 94305, USA; E-Mail: kormond@stanford.edu; 2 Department of Pediatrics, Division of Medical Genetics, Stanford University School of Medicine, 300 Pasteur Drive, Boswell Building A097, Stanford, CA 94304, USA; 3 Irvine School of Medicine, University of California, 1001 Health Sciences Road, 252 Irvine Hall, Irvine, CA 92697, USA; E-Mail: slvernez@gmail.com; 4 Department of Genetics, Stanford University, Mail Stop-5208, Stanford, CA 94305, USA; 5 Department of Sociology, Stanford University, Stanford, CA 94305, USA; E-Mail: mgranovetter@stanford.edu

**Keywords:** genetic testing, direct-to-consumer, social networking, personalized medicine, disclosure of genetic risks

## Abstract

Little is known about how consumers of direct-to-consumer personal genetic services share personal genetic risk information. In an age of ubiquitous online networking and rapid development of social networking tools, understanding how consumers share personal genetic risk assessments is critical in the development of appropriate and effective policies. This exploratory study investigates how consumers share personal genetic information and attitudes towards social networking behaviors. Methods: Adult participants aged 23 to 72 years old who purchased direct-to-consumer genetic testing from a personal genomics company were administered a web-based survey regarding their sharing activities and social networking behaviors related to their personal genetic test results. Results: 80 participants completed the survey; of those, 45% shared results on Facebook and 50.9% reported meeting or reconnecting with more than 10 other individuals through the sharing of their personal genetic information. For help interpreting test results, 70.4% turned to Internet websites and online sources, compared to 22.7% who consulted their healthcare providers. Amongst participants, 51.8% reported that they believe the privacy of their personal genetic information would be breached in the future. Conclusion: Consumers actively utilize online social networking tools to help them share and interpret their personal genetic information. These findings suggest a need for careful consideration of policy recommendations in light of the current ambiguity of regulation and oversight of consumer initiated sharing activities.

## 1. Introduction

The convergence of genomic sequencing technologies, ubiquitous internet connectivity and broad proliferation of social networking tools have contributed to the current context of direct-to-consumer (DTC) genetic testing. Over the past decade, the number of companies that offer genetic testing directly to consumers through the Internet and return personalized genetic information on disease risk, drug response, genetic ancestry, and behavioral traits have proliferated. As genome sequencing costs continue to fall precipitously and the barriers to connecting with others decline, attitudes about sharing personal genetic information may upend long held assumptions about notions of privacy, expertise and genetic exceptionalism. Understanding how and why consumers share personal genetic risk assessments is critical to the development of appropriate and effective evidence-based policies. However, little is known about the motivations and behaviors of these consumers or how they understand and interact with their genetic information when deciding to share results. Key questions for research include: What are consumers’ attitudes towards social networking and sharing of personal genetic information? With whom do consumers share their genetic tests results? How do consumers share their genomic information and what tools do they utilize? This paper discusses findings from a mixed methods study on sharing decisions among individuals, who have purchased DTC personal genetic services and explores consumer experiences and perspectives on social networking and personal genetic information.

A central debate in the growing, yet highly controversial DTC personal genomics industry focuses on who should have access to personal genetic information and under what circumstances. The dominant paradigm for oversight of genetic testing has been informed by genetic exceptionalism—the idea that genetic information is “of *exceptional* ethical significance and worthy of special regulation” [1]. In the exceptionalist view, genetic information imbues a special moral import as it may be instrumental in gaining entree to fundamental biological factors contributing to individual identity and experience. It also holds significance for the ways in which it situates individual identities in relation to one another, creating “molecular families” based on genetic relatedness:

The notion of the molecular family is based on the cultural expectation that a biological entity can determine emotional connections and social bonds—that genetics can link us to each other and somehow preserve a reliable model for a family. Since it is beyond culture, outside of time, DNA seems to be of durable or permanent significance [2].

DTC personal genomic companies such as 23andMe, Inc., which offers personal genotyping on a range of diseases and conditions through the Internet, both build on the special nature of genetic information and at the same time challenges presumed dangers associated with its disclosure outside of the healthcare system [3]. Several researchers have suggested that individuals have a right to obtain genetic information without the prescription and supervision of physicians and other healthcare providers [4,5,6]. Others have criticized DTC personal genetic services as premature and departing from best practices in clinical genetics [7,8,9]. An important primary concern is whether the tests offered to consumers are clinically valid and/or useful [10]. Furthermore, given the serious nature of many of the diseases and traits tested, there is widespread speculation that individuals may misinterpret results and their predictive power, possibly resulting in psychological harm [11,12,13,14,15,16] or needless costly medical follow up [17,18]. 

Only a few studies have been conducted with individuals who actually receive genetic testing and investigate how they interpret and act on their test results. These suggest only modest behavioral changes of relatively short duration [5,19]. A survey based study of 2037 technology and healthcare company employees who were offered personal genetic testing through Navigenics, Inc. concluded that participants did not experience significant pre- or post-test anxiety [20]. Although some studies of the general public indicate that participants anticipate that they would consult with their healthcare providers for help interpreting and applying their results [18,21,22], others studies show that few actually did consult their healthcare providers [19,20,23]. 

Relatedly, the impact of DTC personal genotyping on healthcare services is unclear. Although few studies have investigated this, initial findings seem to indicate that individuals receiving personal genetic information do not increase their use of health care services. The Multiplex Initiative, a research study that enrolled patients who were members of the Henry Ford Health System in Detroit, Michigan, a large managed care organization, studied healthcare usage before and after genetic testing. The study included 1,599 continuously insured adults aged 25–40 years who were offered a multiple genetic susceptibility test for eight common health conditions. Health-care utilization from automated records was compared in 12-month pre- and post-test periods among persons who completed a baseline survey only (68.7%), those who visited a study website but opted not to test (17.8%), and those who chose the multiplex genetic susceptibility test (13.6%). Researchers found that there were no statistically significant differences by group in the pretest use of any common medical tests or procedures associated with four common health conditions. When changes in physician and medical test and procedure use in the post-test period were compared among the groups, no statistically significant differences were observed for any utilization category [24].

However, sharing of personal genetic information goes beyond the healthcare system. DTC personal genomics companies offer consumers an increasingly broad array of services and platforms through which they may share their results with others, including friends, families and online acquaintances and audiences [25]. The personal genomics company 23andMe, Inc. provides web-based tools for allowing consumers to easily grant others access to their 23andMe test results. In addition, 23andMe, Inc. allows consumers to download their raw data, which consumers may elect to share with others through web-based services, social networking sites such as Facebook, or by simple email. Genomera, Inc. is one example of a growing number of companies that allows consumers to upload their personal genetic data for “commercial or academic studies at Internet scale” in an effort to crowdsource data for “do-it-yourself” genetic health studies [26]. While these various venues are available to consumers, little empirical research has probed how consumers of DTC personal genetic testing engage in these sharing practices and their attitudes underpinning these decisions. 

## 2. Methods

A web-based questionnaire was administered to consumers of 23andMe personal genetic testing services as part of a mixed methods study that included in-depth interviews and focus groups. To recruit consumers of DTC personal genetic testing services, information about this study was published in *The Spittoon*, the 23andMe blog that is emailed directly to 23andMe consumers. Announcements of the study were also placed on Facebook and in ancestry and genetic testing blogs in 2010 with information on how to inquire about enrollment. Information was also published on the independent study website [27].

Potential participants were directed to contact our research team at Stanford University and were limited to individuals who had purchased genetic testing services from 23andMe, Inc. and who were 18 years or older at the time of enrollment. Only registered users of 23andMe, Inc., who had no affiliation with the company and who enrolled in the larger study were invited to take the survey. The survey took an estimated 15–30 min to complete. Individuals engaged in the survey following participation in in-depth face-to-face or telephone interviews lasting approximately 90 min to 2 h. The results reported in this manuscript focus on the web-based survey responses. No 23andMe employees or company affiliates were involved in recruiting specific participants, nor in any other aspects of enrollment, study design, data collection nor analysis. Informed consent was obtained from each participant. The study was approved by Stanford University School of Medicine Office of Human Subjects and institutional review boards. 

Online surveys were administered using the web based survey tool, SurveyMonkey [28]. Survey questions explored: (1) general attitudes towards and practices of social networking and online interactions; (2) experience with DTC personal genetic testing and sharing of genetic test results and data; (3) demographic information (age, gender, race, education level, occupation, and access to health care). Survey questions included statements accompanied by a 5-point scale ranging from “strongly disagree” to “strongly agree.” The survey instrument was created through an iterative process, pretested by the research team, and revised accordingly before administered to participants. Descriptive statistics were calculated to summarize participants’ responses by all and by user status. A chi-square test was performed to examine the relationship between answers to different questions. *p*-values of 5% or less were considered significant. Data on chi-square analyses are not shown.

## 3. Results and Discussion

### 3.1. Demographic Characteristics

Participants ranged in age from 23 to 72 years (mean: 43.6, SD 14.2 years). The majority (80.3.%) identified as white or Caucasian, 3% reported Asian or Asian American identity, 6.3% identified as Hispanic or Latino, 1.7% African or African American and 5% selected Other. Participants were allowed to identify with multiple racial and ethnic categories, which account for the percentages totaling more than 100%. Participants who chose the “other” category wrote in identities such as “black/white”, “Brazilian”, “Jewish” and “New World Hispanic.” The median household income was in the range of $50,000 to $100,000 although approximately one-third of participants (31.6%) earned less than $50,000. The overwhelming majority (91.7%) graduated from college although nearly half (47.5%) reported no formal educational background in genetics. A small number of participants (13.6%) reported having a degree in genetics or having worked professionally in the field. Similarly, a minority of participants (18.3%) reported working in some health related occupation. Most participants rated their health as either excellent or very good (75%) and the majority indicated having health insurance (80.6%). Most reported having a regular physician (78.3%) and a regular health check-up within the last year (70%) (Table 1). In summary, our study cohort reflects a predominantly white, highly educated, middle income population with good access to healthcare. These demographics are similar to reports from other empirical studies of consumers [19,29] and market statistics from 23andMe, Inc. 

**Table 1:** Sample descriptive statistics for N = 80 study participants.

Demographic and health information seeking characteristics	Sample Characteristic
Gender (% female)	50.0
Age (year)	
Mean	43.6 ± 14.2
Range	23–72
White race (% of Sample)	80.3%
**Household Income ($)**	
**Median**	**50 k–99 k**
<50 k	31.6%
50 k–99 k	29.8%
100 k–149 k	22.8%
150 k–199 k	3.5%
200 k–249 k	1.8%
250 k–299 k	3.5%
>300 k	7.0%
**Education**	
**Median**	**Masters degree**
High School or GED	0.0%
Some college	5.0%
2-year college graduate	5.0%
4-year college graduate	33.3%
Master’s degree	31.7%
Professional Degree	21.7%
Other	3.3%
**Background in genetics**	
**Median**	**High School Course**
None	47.5%
High school course	20.3%
College course(s)	22.0%
Degree in genetics	1.7%
Professional work in genetics	11.9%
**Health-related occupation (% of sample)**	**18.30%**
**Self-reported health status rating**	
**Median**	**Very good**
Excellent	31.7%
Very good	43.3%
Good	21.7%
Poor	3.3%
Very Poor	0.0%
**Insurance Status (% of sample insured)**	**80.6%**
**Regular Physician (% of sample who has a regular physician)**	**78.3%**
**Last time participant had a routine check-up**	
**Median**	**within the last year**
Within the past year	70.0%
Within the past two years	18.3%
Within the past 5 years	6.7%
5 or more years ago	5.0%
**How much did you pay for your 23and Me Kit?**	
**Median**	**$199**
Free	5.0%
$99	36.7%
$199	16.7%
$399	16.7%
$999	3.3%
Other	21.7%

Similar to research reported by McGuire *et al*. [29], the most commonly cited reason among those who signed up for DTC personal genetic services was to satisfy a general curiosity about one’s genetic makeup (51.9%). This was followed by interests in genetic ancestry and then disease risk, carrier status, and pharmacogenomic information. Participants expressed a general interest in genetics, as opposed to specific concerns about conditions known in one’s own or family’s medical history.

### 3.2. Social Networking and Sharing of Genetic Information

Personal genomic companies like 23andMe, Inc. contribute to the growing infrastructure of online connectivity around health by providing tools like *Genome Sharing* and comparison tools that allow consumers to easily share their 23andMe genetic profile with other 23andMe consumers through the company platform. Probing the social networking characteristics of DTC genetic testing consumers, our study suggests a consumer profile that is relatively active on social networking sites and shares genetic information through a range of modalities, including the popular social media platform Facebook. 

Study participants reported active participation on social networking sites (Table 2). The most common site frequented by participants was Facebook (96.6%) followed by LinkedIn (67.2%) and Twitter (55.2%). Nearly a quarter of respondents reported participating on additional sites and logging onto at least one social networking site 2–3 times a day. Of the participants who identified themselves as Facebook users, 54.7% reported having over 150 Facebook friends. Given that DTC personal genotyping services are offered nearly exclusively online, it is perhaps not surprising that consumers of DTC personal genetic testing also participate in social media, particularly as advertisements for services are often posted on these pages. The use of social networking as a medium for seeking health information may also explain the current landscape of DTC personal genomics.

**Table 2:** Sharing behaviors among consumers.

Social Networking and Personal Genetic Testing	
**Shared personal genetic information on Facebook**	45.0%
**Downloaded their raw data**	52.7%
**Verbally shared genetic results**	85.5%
**Shared 23andMe Username and Password**	29.1%
**Emailed genetic results to others**	52.7%
**Number of people participants met or reconnected with by sharing personal genetic test results**	
**Median**	**>10**
No one	20.8%
1	1.9%
<5	13.2%
5–10	13.2%
>10	50.9%
**Number of people participants consulted about genetic testing results**	
**Median**	**<5**
No one	14.8%
1	14.8%
<5	40.7%
5–10	13.0%
>10	16.7%

Most participants (66.7%) reported that they have gone online (23andMe website, Facebook and other platforms) for the purpose of finding others with similar health concerns. The majority of participants also reported connecting with others through the 23andMe website, and in particular, the company’s online community groups (70%) and a large proportion of participants (45%) reported sharing their personal genetic information on Facebook. When asked how many people they met or reconnected with by sharing their genetic test results, 50.9% reported 10 or more people. Although 14.8% indicated that they did not consult others about their genetic test results, 40.7% reported consulting five or more individuals for help interpreting and/or acting upon their genetic test results. 

Nearly all participants shared their personal genetic information with family members (98.3%) and friends (81.7%). A large proportion of participants also shared with other 23andMe consumers (76.7%) who they met online as compared to 56.6% who shared their personal genetic information with a health professional. The percentage of our participants that reported that they shared their results with a health professional is comparable to results reported from the study conducted by McGuire *et al*. [29] of Facebook users who had previous experience with DTC personal genetic testing (53%) yet higher than reported by Bloss *et al*. [30] (39.5%). However, when participants asked who they consulted for help with interpreting their genetic information, the most commonly reported resources were Internet websites (70.4%) followed by 23andMe Help (53.7%). Only 22.7% reported consulting a healthcare provider for help. 

This study suggests that consumers of DTC personal genetic testing look outside of the healthcare system to seek expertise in interpreting their results. Similar to the Multiplex Initiative Study that returned genetic tests results to healthy adults [28], our study shows that consumers of DTC personal genetic testing services were more likely to consult online sources than their healthcare providers to make sense of their personal genetic information. This may be explained in part by the finding that the majority of participants reported that their healthcare provider had limited ability to understand and insufficient expertise to interpret their results (77.8%). 

When asked how they shared their personal genetic information, a little more than half of participants (52.7%) reported downloading their raw data in order to share. Similarly, 52.7% reported emailing information and 29.1% gave their 23andMe log-in and password information to others to access their accounts. Nearly two-thirds of participants (61.6%) reported managing 23andMe accounts other than their own, the majority of which were for other family members (86.5%) and friends (13.5%). 

### 3.3. Attitudes towards Privacy

Not surprisingly, most participants reported being comfortable with online interactions (Table 3). For example, nearly all (98.1%) reported ease with conducting financial transactions online. A large majority (85.2%), however, believed that privacy is never guaranteed when online and approximately half (51.8%) believed that the privacy of their personal genetic information may be breached in the future. However, most (64.8%) felt confident that 23andMe would not share their information with others without their permission. A small number (11.3%) reported concern that their employer would learn of their personal genetic test results although more (30.2%) reported concern that their personal genetic test results would have negative implications on their ability to obtain health, life and/or disability insurance. The majority of participants (81.5%) believed that sharing their personal genetic information for biomedical research is an important individual responsibility.

**Table 3:** Attitudes towards privacy among study participants.

	Strongly/Agree	Neutral	Strongly/Disagree
I am comfortable shopping and/or conducting financial transactions online.	98.10%	0.00%	1.90%
I have global concerns about confidentiality of personal data.	50.00%	18.50%	31.50%
I believe that privacy is never guaranteed when interacting online.	85.19%	9.26%	5.56%
I am concerned that my employer will learn about my personal genetic test results.	11.30%	17.00%	71.70%
I believe that the privacy of my personal genetic information may be breached in the future.	51.80%	24.10%	24.10%
I do not want to learn genetic information that could potentially jeopardize my family’s confidentiality.	17.00%	22.60%	60.40%
I am concerned that obtaining my personal genetic information will have negative implications on my ability to obtain health, life and/or disability insurance.	30.19%	15.09%	54.72%
I feel confident that my personal genetic information will NOT be shared with others without my permission.	64.80%	22.20%	13.00%
I believe that sharing my personal genetic information for biomedical research is an important individual responsibility.	81.50%	11.10%	7.50%

The easy transfer of personal genetic information either through email or uploading of raw data onto social media platforms begs the question of whether consumers of DTC personal genomics have concerns over privacy. Our study results indicate that the majority does not assume that privacy is guaranteed online and that many predict that their personal genetic information may be disclosed in the future. However, it should be noted that concerns about privacy did not deter their purchase of DTC genomic testing nor their participation in sharing or social networking. Studies of genetic risk perception suggest a relationship between risk intolerance and psychological distress [20]. Our study suggests that the participants perceived risks to their privacy, but were highly tolerant, which may be explained by their relatively healthy status and that the majority were insured. It may also be the view of consumers that a risk to privacy is the price they pay for access to personal genetic information. Further research on attitudes towards social networking and genetic privacy in the context of risk perception is needed.

Our participants reported routinely utilizing the Internet as a resource for health information and searching for other individuals who may have similar health concerns. Such activity has been identified as a feature of what many refer to as Health 2.0, where the ubiquity of personal computers and easy access to the Internet allows individuals to create connections and community around common interests and concerns. There have been indications of disease focused online congregations such as *PatientsLikeMe* where individuals share information about their conditions and healthcare experiences. This has been particularly true for patients affected by rare diseases where online advocacy groups have increased ability of affected individuals to share experiences and support, and to raise public awareness [29,31]. As such, the value of sharing among consumers of DTC personal genetic testing may be in the quality of connections that personal genetic information can create and the potential of large datasets. The perceived value of these social formations may play significantly in the algorithms used by consumers in weighing risks to their privacy and the benefit of sharing.

### 3.4. Limitations of the Study

Our study is limited in its scope to one DTC personal genomics company. 23andMe, Inc. was chosen because of its high level of recognition within the DTC personal genomics market [32] and the broad array of genetic tests offered by the company. Other companies that require involvement of healthcare providers in the purchasing of testing, such as Navigenics and Pathway Genomics, were deemed as not distinctively direct-to-consumer and were not included in the study. In addition, consumers were not randomly selected and may be selective of individuals apt to share information and participate in research or who have an exceptional interest in, and thus perspective on, personal genetics. The majority of our data is based on self-reported information and as indicated, reflects a particular demographic profile that does not reflect the general U.S. population. 

## 4. Conclusions

Our study addresses the current gap in published research on consumer use of DTC personal genomic services, attitudes towards social networking, and sharing experiences of personal genomic information. Having recruited individuals who purchased DTC personal genetic testing on their own, this study provides a unique opportunity to probe the attitudes and experiences of actual consumers. Our study suggests that consumers of DTC personal genetic testing services actively use social networking sites and share their personal genetic information with others through the Internet. The turn to online sources for help interpreting genetic test results point to the need for further research on the meaning of “expertise” in the context of social networking and DTC personal genomics, particularly given the relatively low level of consultation with healthcare providers. Further research is needed to identify what counts as expertise to consumers, what type of information they are receiving and how consumers are applying the advice they receive. 

Furthermore, given the ambiguity in legal protection of personal genetic information, the sharing of personal genetic information warrants focused discussion of the potential implications of such behaviors for individual privacy. This is particularly important given that our study shows that consumers are more likely to consult friends and “Internet acquaintances” through social networking platforms about their genetic test results than their own healthcare providers. Consumer concerns over privacy concerns did not deter their purchase of DTC personal genetic testing nor their participation in sharing or social networking. Our study suggests that risk perception is not tantamount to risk tolerance, as study participants seem to value access to their personal genetic information over issues of confidentiality and privacy. In particular, study findings suggest a need to reframe how we think about and potentially regulate access and dissemination of genetic information as consumers increasingly utilize social networking platforms in the context of DTC genetic testing.

In the current ambiguous regulatory landscape, consumers’ complex behaviors and attitudes suggest a need for thoughtful consideration of their legal and social implications. Online networking is essentially unregulated. A looming question is whether commercial providers have a codified duty beyond their sales contract with their consumers in the absence of a healthcare provider relationship. As consumers take the option of downloading their raw data and choose to email and upload their genomic information onto social networking platforms, consumers require best practices from the industry for guiding sharing behaviors. The stakes for such issues are raised as social networking becomes an increasingly attractive vehicle for health information exchange [33] and companies chip away at a paradigm founded on genetic exceptionalism and extend the offer of consumer control over personal genetic information. Policy that takes into account consumer decisions on social networking and sharing must be incorporated into the current debate on the ethical, legal and social implications of DTC personal genomics.
